# Supplementary material for: Splice-Junction-Based Mapping of Alternative Isoforms in the Human Proteome
Source: Cell Rep. Author manuscript; Available in PMC 2020 Jan 15. (PMC6961840; doi:10.1016/j.celrep.2019.11.026)
Supplement: 2 [file NIHMS1546469-supplement-2.zip › DF1/PXD010154/Heart/Heart_46_TTN_EAPPAKVPEEPKR.pdf]

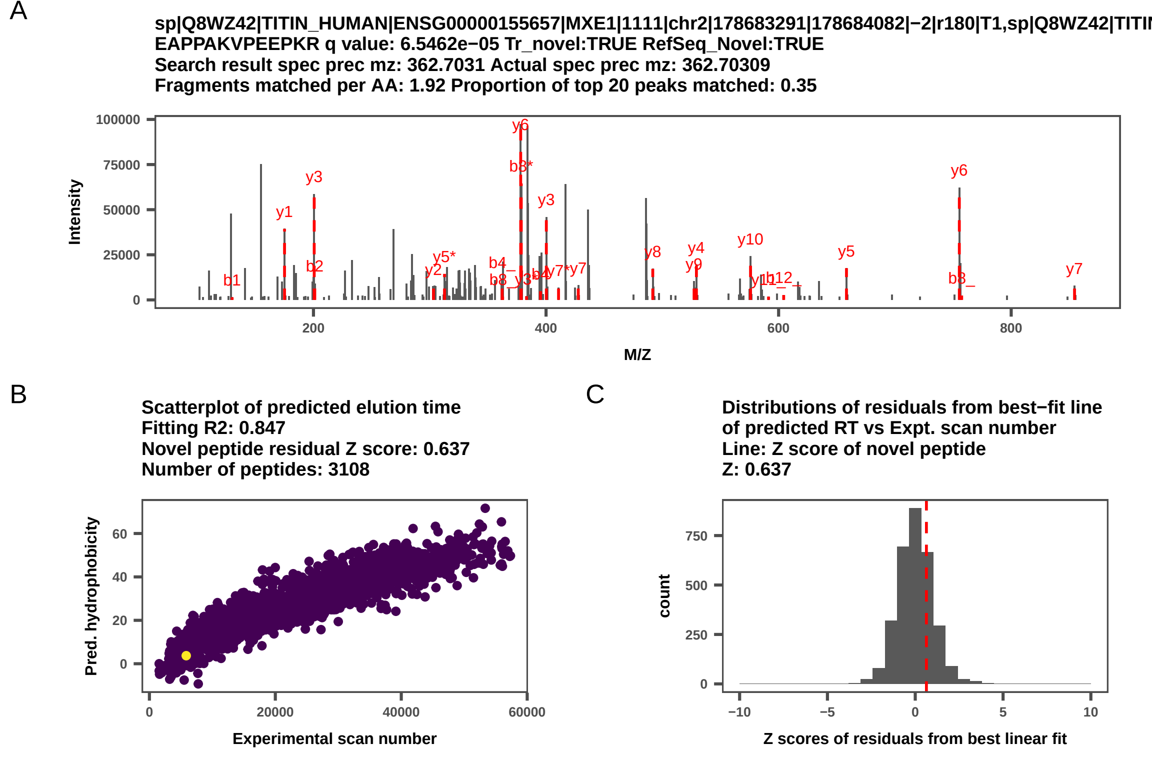
A
sp|Q8WZ42|TITIN_HUMAN|ENSG00000155657|MXE1|1111|chr2|178683291|178684082|−2|r180|T1,sp|Q8WZ42|TITI
EAPPAKVPEEPKR q value: 6.5462e−05 Tr_novel:TRUE RefSeq_Novel:TRUE
Search result spec prec mz: 362.7031 Actual spec prec mz: 362.70309
Fragments matched per AA: 1.92 Proportion of top 20 peaks matched: 0.35
Intensity
100000
75000
50000
25000
0
M/Z
200
400
600
800
y6
b8*
y3
y1
y3
b2
b1
y5*
y2
b4
y7*
y7
y8
y4
y9
y10
y12
y5
y6
y7
B
Scatterplot of predicted elution time
Fitting R2: 0.847
Novel peptide residual Z score: 0.637
Number of peptides: 3108
Pred. hydrophobicity
60
40
20
0
0
20000
40000
60000
Experimental scan number
C
Distributions of residuals from best−fit line
of predicted RT vs Expt. scan number
Line: Z score of novel peptide
Z: 0.637
count
750
500
250
0
−10
−5
0
5
10
Z scores of residuals from best linear fit
